# Normal and equivolumetric coordinate systems for cortical areas

**DOI:** 10.1016/j.mex.2024.102689

**Published:** 2024-04-01

**Authors:** Laurent Younes, Kwame S. Kutten, J. Tilak Ratnanather

**Affiliations:** a Department of Applied Mathematics and Statistics, Johns Hopkins University, Baltimore, MD, USA; b Department of Biomedical Engineering, Johns Hopkins University, Baltimore, MD, USA

**Keywords:** Normal Equivolumetric Coordinate System, Shape analysis, Medical imaging, Cortical thickness, Equivolumetric coordinates, Bok's hypothesis

## Abstract

We describe coordinate systems adapted for the space between two surfaces, such as those delineating the highly folded cortex in mammalian brains. These systems are estimated in order to satisfy geometric priors, including streamline normality or equivolumetric conditions on layers. We give a precise mathematical formulation of these problems, and present numerical simulations based on diffeomorphic registration methods, comparing them with recent approaches.

Our method involves

• Diffeomorphic registration of inner and outer folded folded surfaces.
• Followed by equivolumetric reparametrization of layers to yield coordinate system.

## Graphical abstract

**Figure:**
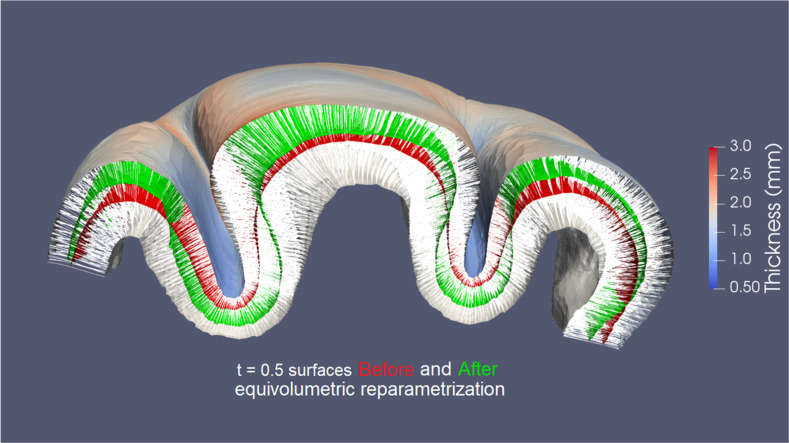

Specifications table

**Table:** 

Subject area:	Mathematics
More specific subject area:	Brain Cortical thickness and depth
Name of your method:	Normal Equivolumetric Coordinate System
Name and reference of original method:	Name: Normal Coordinate System Reference: J. T. RATNANATHER, S. ARGUILLÈRE, K. S. KUTTEN, P. HUBKA, A. KRAL and L. YOUNES, *3D normal coordinate systems for cortical areas*, In. Mathematics of shapes and applications, World Scientific, 2020, pp. 167–179 Link: https://doi.org/10.1142/9789811200137_0007
Resource availability:	Code: https://bitbucket.org/laurent_younes/py-lddmm/src/master/

## Background

Anatomical regions of interest extracted from 3D biomedical imaging data often appear as volumes separated by “upper” and “lower” surfaces. These include, in particular, the highly folded cortical regions or areas of the mammalian brain. This paper focuses on methods that parametrize such volumes, using coordinate systems that are naturally aligned with the encompassing surfaces. Our contributions are, on one hand, to formalize a notion of laminar coordinate systems and, on the other hand, discuss within this formalism the concept of equivolumetric coordinates [1,2], providing an interpretation of two similar methods [3,4] and introducing a new one based on diffeomorphic registration methods.

**Fig. 1:**
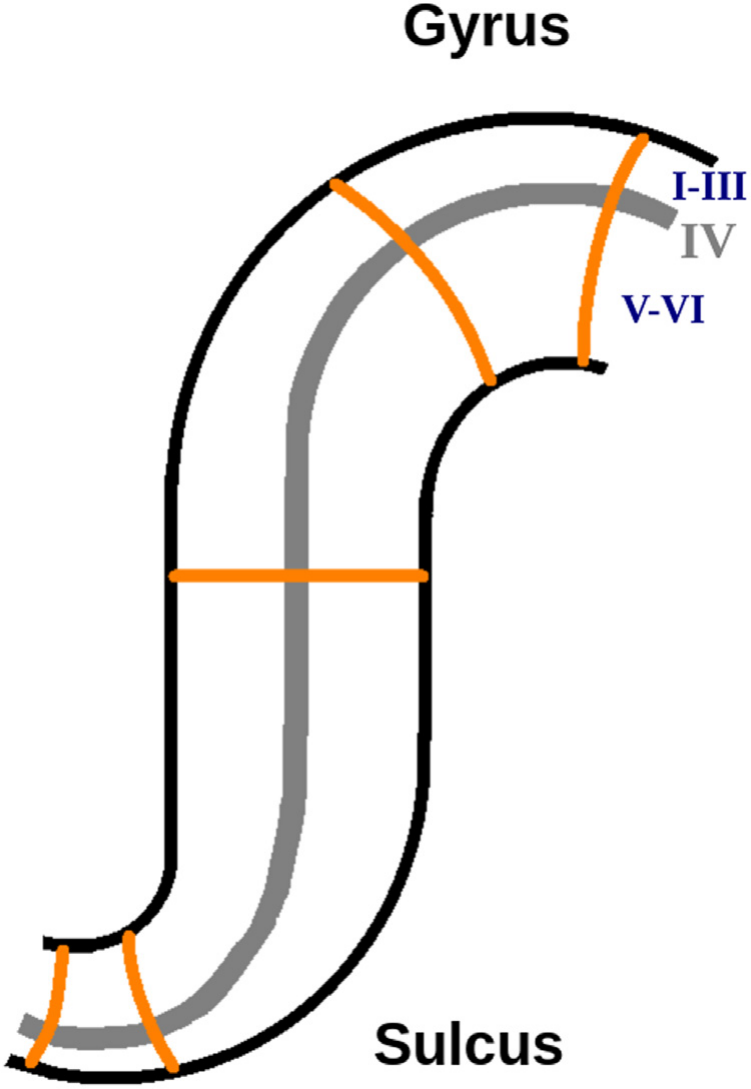
Idealisation of Bok's equivolumetric hypothesis for the layers in the folded cortex showing that the deep layers (V-VI) are thin in the sulcus and thick in the gyrus respectively characterized by regions of positive and negative inward curvature and conversely for the upper layers (I-III).

The elegant laminar structure of a typical cortical area [5,6] is summarized as follows. The folding of the area serves to maximize its surface area in a confined cranial space. The neural tissue (grey matter) within the area contains mostly neuronal cell bodies and unmyelinated fibers. Cortical areas (which number in the hundreds in the human brain) are connected via white matter containing axonal, usually myelinated, fibers. Each cortical area is composed of fundamental units called cortical columns that traverse vertically from the white matter to the surface just below the pial matter. Finally, the cortical area is composed of six layers which are stacked horizontally on top of each other. Bok [1,2] observed that to maintain the laminar structure in highly folded regions thin layers in one part became thicker in another part. This observation led to the hypothesis that the cortex satisfies an equivolumetric property, illustrated in Fig. 1, which provides the theoretical motivation of this paper.

## Method details

Our method is described below. Section 1 introduces formal definitions of laminar coordinates and describes methods for constructing them. Section 2 describes how Bok's equivolumetric hypothesis can be implemented.

### Laminar coordinate systems and thickness

#### Notation

**Fig. 2:**
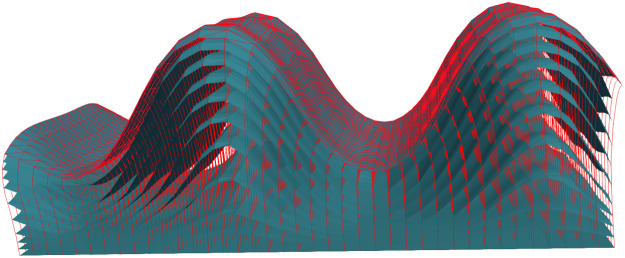
A laminar coordinate system between two surfaces, represented by the red scaffold.

We introduce some mathematical notation in order to describe 3D coordinate systems parametrizing an open space between two surfaces. We will call “laminar coordinate system” (see Fig. 2 for an example) a special case of a *foliation* of this open set, with two special leaves provided by the two surfaces.

More precisely, let $S_{0}$ and $S_{1}$ be 2D submanifolds (surfaces) in $R^{3}$, such that $S_{0}\cap S_{1}=\emptyset$. Assume that these surfaces are bounded, and that they are either compact (e.g., spheres), or surfaces with boundaries (e.g., disks). A laminar coordinate system between $S_{0}$ and $S_{1}$ is a $C^{1}$ embedding $\psi :\left[0,1\right]\;\times S_{0}\to R^{3}$ such that ψ(0,x)=x for all $x\in S_{0}$ and ψ(1,·) maps $S_{0}$ onto $S_{1}$. The embedding assumption requires that (i) ψ in one-to-one, (ii) $\partial_{x}\psi \left(t,x\right)$ (the differential of ψ(t,·) at x) can be extended by continuity to the whole set $\left[0,1\right]\;\times S_{0}$ and is an invertible linear mapping, and (iii) the inverse mapping defined on $\bar{\Omega }=\psi \left(\left[0,1\right]\times S_{0}\right)$ is continuous.

The surfaces $S_{t}=\psi \left(t,S_{0}\right)$ are the leaves of the foliation, and will be referred to as “layers” while the curves $\gamma_{x}\left(t\right)=\psi \left(t,x\right)$, t∈[0,1] will be referred to as “streamlines.” These layers and streamlines are shown as blue surfaces and vertical red lines respectively in Fig. 2. The set $\bar{\Omega }=\psi \left(\left[0,1\right]\times S_{0}\right)$ is the closure of an open subset Ω of $R^{3}$ defining the space between the two surfaces.

In addition, a coordinate system defined as such provides a notion of “thickness” of Ω at $x\in S_{0}$, given by the length of the streamline starting at x, namely,

$$\theta \left(x\right)=\int_{0}^{1}\left|\partial_{t}\psi \left(t,x\right)\right|\;dt.$$

#### Coordinate systems

Several methods for building coordinate systems have been described in prior research. Two classes of these approaches are level set methods (such as Jones et al. [7] and Waehnert et al. [4]) and those based on diffeomorphic volume mapping (such as Das et al. [8] or Fischl and Sereno [9]). As these methods (which are further described in supplementary material) are volumetric, they work on 3D regions rather than directly on surfaces, which are represented either as level sets or as boundaries of open sets. Hence, they are, in particular, not well adapted to analyze regions delimited by open surfaces. So following [10], a version of the Large Deformation Diffeomorphic Metric Mapping (LDDMM) algorithm (Beg et al. [11]) adapted to surface mapping was combined with normality constraints in order to estimate laminar coordinate systems. This is now described in some detail.

Assume that $S_{0}$ is parametrized over a bounded open subset $M\subset R^{2}$ (or more generally over a 2D manifold M with or without boundary) in the form $S_{0}=q_{0}\left(M\right)$, where $q_{0}$ is an embedding of M into $R^{3}$. The LDDMM surface registration algorithm solves an optimal control problem minimizing, over all time-dependent vector fields in a reproducing kernel Hilbert space V,

(1) $$\int_{0}^{1}\|\|v\left(t\right)\|{\|}_{V}^{2}\;dt+D\left(q\left(1,M\right),S_{1}\right)$$

subject to $q\left(0\right)=q_{0}$ and $\partial_{t}q\left(t\right)=v\left(t\right)\circ q\left(t\right)$. Here, D is a reparametrization-invariant discrepancy measure between (unparametrized) surfaces. Several versions of this cost function have been introduced, based on representations of surfaces as currents [12], varifolds [13,14] or normal cycles [15].

Assume that $S_{0}$ and $S_{1}$ are triangulated surfaces and that the cost function D is replaced by a discrete approximation, still denoted D. Then, the optimization problem can be reduced to one tracking explicitly the evolution of the vertices of the triangulation, using the reproducing kernel of V denoted as K. This kernel is a matrix-valued function of two variables $x,y\in R^{3}$ such that, for all $\alpha ,y\in R^{3}$, the vector field x↦K(x,y)α belongs to V and for all v∈V,

$${\langle v\;\;K\left(\cdot ,y\right)\alpha \rangle }_{V}=\alpha^{T}v\left(y\right)$$

where the left-hand side denotes the inner product in V.

Denote as $q_{0}=\left(q_{0}\left(1\right),\ldots ,q_{0}\left(N\right)\right)$ the vertices of $S_{0}$. The reduced problem is expressed in terms of evolving vertices q(t)=(q(t,1),…,q(t,N)) and vectors α(t)=(α(t,1),…,α(t,N)), t∈[0,1], minimizing (letting S(t) denote the triangulated surface with vertices q(t) and same topology (faces) as $S_{0}$)

$$\int_{0}^{1}\sum_{k,l=1}^{N}\alpha {\left(t,k\right)}^{T}K\left(q\left(t,k\right),q\left(t,l\right)\right)\alpha \left(t,l\right)\;dt+D\left(S\left(1\right),S_{1}\right)$$

subject to $q\left(0\right)=q_{0}$ and

$$\partial_{t}q\left(t,k\right)=\sum_{l=1}^{N}K\left(q\left(t,k\right),q\left(t,l\right)\right)\alpha \left(t,l\right)$$

for k=1,…,N. Moreover, the optimal vector field at time t is given by

(2) $$v\left(t,\cdot \right)=\sum_{l=1}^{N}K\left(\cdot ,q\left(t,l\right)\right)\alpha \left(t,l\right).$$

The interest of this formulation is that the trajectories t↦q(t,k) for k=1,…,N directly provide the streamlines starting from the vertices $q_{0}\left(k\right)$, k=1,…,N of the triangulation of $S_{0}$.

**Fig. 3:**
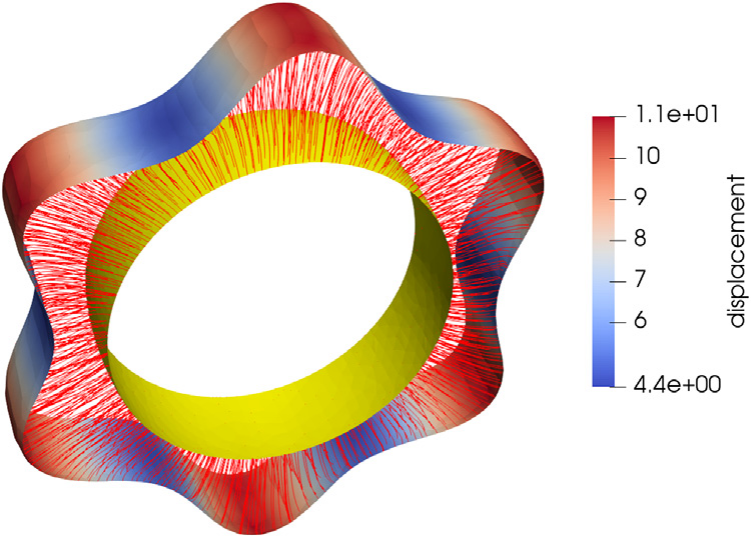
Synthetic data for thickness estimation between an inner ring of fixed radius and an outer one of variable radius. The colors on the outer ring show thickness values increasing from blue to red.

This algorithm is modified by imposing a constraint ensuring that these streamlines are perpendicular to the evolving layers [10]. In the continuous setting, where, for each t∈[0,1] q(t,·) is defined on the manifold M, this constraint can be formulated as $v\left(t,q(t,s)\right)=\lambda \left(t,s\right){\vec{n}}_{S(t)}\left(q(t,s)\right)$, t∈[0,1], s∈M. Here ${\vec{n}}_{S}\left(x\right)$ denotes the (positively oriented) unit normal to an oriented surface S at x∈M, and λ(t,s) is a scalar that we assume to be non-negative (with a proper orientation of S(t) to prevent the trajectories from backtracking). The constraint is implemented in the equivalent form

$$\sqrt{v{\left(t,q\left(t,s\right)\right)}^{T}v\left(t,q\left(t,s\right)\right)}-{\vec{n}}_{S\left(t\right)}{\left(q\left(t,s\right)\right)}^{T}v\left(t,q\left(t,s\right)\right)=0,$$

that is discretized on the (evolving) triangulation of S(t). The resulting constrained optimization problem is solved using an augmented Lagrangian method [16] with each gradient descent step implemented using a limited-memory Broyden–Fletcher–Goldfarb–Shanno (BFGS) method (for details of methods using LDDMM with constraints, see [17]). Fig. 3 illustrates a synthetic example of how the thickness map can be estimated using this method.

Note that the LDDMM algorithm implicitly provides a flow of diffeomorphisms on the whole space $R^{3}$, given by solution of $\partial_{t}\varphi \left(t,\cdot \right)=v\left(t,\varphi \left(t,\cdot \right)\right)$.

Even if we will not use it in the following, it is interesting to notice is that this method generally provides a level-set formulation of the laminar coordinates. Indeed, under the mild assumption that v(t,q(t,s)) never vanishes, the function $\psi :\left[0,1\right]\;\times S_{0}\to R^{3}$ defined by $\psi \left(t,q_{0}\left(s\right)\right)=q\left(t,s\right)$ is an immersion. In practice, this mapping is in addition one-to-one and one can define without ambiguity a scalar function F on a domain sandwiched by the surfaces $S_{0}$ and $S_{1}$ (or, more precisely, by $S_{0}$ and $S\left(1\right)≃S_{1}$), by

F(q(t,s))=t

for s∈M. By construction, the streamlines are perpendicular to the level sets of this function.

### Equivolumetric coordinates

#### Strict condition

As mentioned above, Bok's hypothesis requires that the cortical layers satisfy an equivolumetric constraint. Define a “cortical tube” as the volume delimited by the cortical columns stemming from the inner (grey-white) matter surface to the outer (pial) surface. The hypothesis requires that the volume delimited by the intersection of cortical tubes and cortical surfaces remains roughly constant when the base patch is “translated” along the inner surface. We want to formalize this into a definition of “equivolumetric laminar coordinates.”

Consider a laminar coordinate system $\psi :\left[0,1\right]\;\times S_{0}\to R^{3}$. For $x\in S_{0}$, consider a small surface element $\delta S_{0}$ located at x and the infinitesimal tube $\psi (\left[0,1\right]\;\times \delta S_{0})$. Introduce a local chart $m:U\to \delta S_{0}$ on $\delta S_{0}$, where U is an open subset of $R^{2}$. Let $\psi_{m}\left(t,\alpha ,\beta \right)=\psi \left(t,m(\alpha ,\beta )\right)$. Then the volume of the tube between layers $t_{0}$ and $t_{1}$ is given by

$$\int_{t_{0}}^{t_{1}}\int_{U}\text{det}\left(\partial_{t}\psi_{m},\partial_{\alpha }\psi_{m},\partial_{\beta }\psi_{m}\right)\;d\alpha \;d\beta \;dt$$

$$=\int_{t_{0}}^{t_{1}}\int_{\delta S_{0}}\partial_{t}\psi {\left(t,x\right)}^{T}\vec{n}\left(t,x\right)\sigma \left(t,x\right)d\text{vo}l_{S_{0}}\left(x\right)dt.$$

Here, we have used the notation $\vec{n}\left(t,x\right)={\vec{n}}_{S(t)}\left(\psi (t,x)\right)$. We also have denoted by $d\text{vo}l_{S_{0}}$ the volume form on $S_{0}$, which is given, in the local chart, by $|\partial_{\alpha }m\times \partial_{\beta }m|d\alpha d\beta$, and by σ(t,x) the surface Jacobian induced by $\psi \left(t,\cdot \right):S_{0}\to S\left(t\right)$ (infinitesimal ratio of area), defined in the chart by

$$\frac{\left|\partial_{\alpha }\psi_{m}\times \partial_{\beta }\psi_{m}\right|}{\left|\partial_{\alpha }m\times \partial_{\beta }m\right|}\;.$$

One has the following result that describes the evolution of σ as a function of t.

Proposition 1 *Let*$w:\Omega \mapsto R^{3}$*be defined by*$w\left(\psi (t,x)\right)=\partial_{t}\psi \left(t,x\right)$*for all*$\left(t,x\right)\in \left[0,1\right]\;\times S_{0}$*. Define*$\vec{N}:\Omega \mapsto R^{3}$*by*$\vec{N}\left(\psi (t,x)\right)=\vec{n}\left(t,x\right)$*and decompose*w*in the form*

$$w\left(y\right)=\rho \left(y\right)+\zeta \left(y\right)\vec{N}\left(y\right),$$

with $\rho \left(y\right)⊥\vec{N}\left(y\right)$ for all y∈Ω. Let $\rho_{t}$ denote the restriction of ρ to S(t). One has

(3) $$\sigma^{-1}\partial_{t}\sigma =\left(\text{di}v_{S\left(t\right)}\rho_{t}-2\zeta H_{S\left(t\right)}\right)\circ \psi$$

where $\text{di}v_{S(t)}$ is the divergence operator on S(t) and $H_{S(t)}$ the mean curvature on the same surface.

**Fig. 4:**
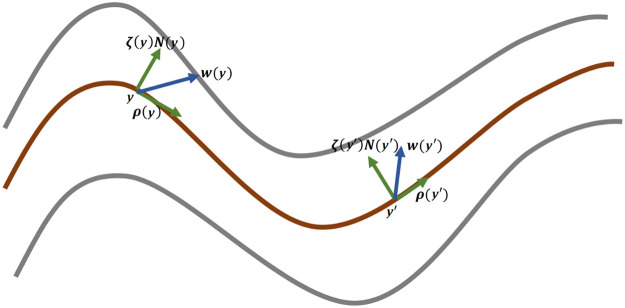
Illustration of the notation used in Proposition 1. The evolution of intermediate surfaces (red) is captured by a vector field w that decomposes into a tangential part ρ and a normal part ζN.

The notation used in this proposition is illustrated in Fig. 4. Recall that the divergence operator of a vector field ρ on a surface S is (for p∈S)

(4) $$\text{di}v_{S}\left(\rho \right)\left(p\right)=e_{1}^{T}D\rho \left(p\right)e_{1}+e_{2}^{T}D\rho \left(p\right)e_{2}$$

where $e_{1},e_{2}$ is any orthonormal basis of $T_{p}S$ (the tangent space to S at p). Using the same basis, the mean curvature is given by

(5) $$-2H_{S}\left(p\right)=e_{1}^{T}D{\vec{n}}_{S}\left(p\right)e_{1}+e_{2}^{T}D{\vec{n}}_{S}\left(p\right)e_{2}.$$

When convenient, will use the notation: $H\left(t,x\right)=H_{S(t)}\left(\psi (t,x)\right)$ to represent the mean curvature along a streamline.

Proof We make the computation in a chart $m:U\subset R^{2}\to S_{0}$ and let

$$J\left(t,\alpha ,\beta \right)=\left|\partial_{\alpha }\psi_{m}\times \partial_{\beta }\psi_{m}\right|$$

so that σ(t,m(α,β))=J(t,α,β)/J(0,α,β). Then

$$\partial_{t}J={\left(\partial_{t}\partial_{\alpha }\psi_{m}\times \partial_{\beta }\psi_{m}\right)}^{T}{\vec{n}}_{m}+{\left(\partial_{\alpha }\psi_{m}\times \partial_{t}\partial_{\beta }\psi_{m}\right)}^{T}{\vec{n}}_{m}$$

with

$${\vec{n}}_{m}\left(t,\alpha ,\beta \right)=\vec{n}\left(t,m\left(\alpha ,\beta \right)\right)=\frac{\partial_{\alpha }\psi_{m}\times \partial_{\beta }\psi_{m}}{\left|\partial_{\alpha }\psi_{m}\times \partial_{\beta }\psi_{m}\right|}.$$

Let $e_{\alpha }=\partial_{\alpha }\psi_{m}$, $e_{\beta }=\partial_{\beta }\psi_{m}$. Note that, letting $w_{m}\left(t,\alpha ,\beta \right)=w\left(\psi_{m}\left(t,\alpha ,\beta \right)\right)$, one has

$$w_{m}\left(t,\alpha ,\beta \right)=\partial_{t}\psi_{m}\left(t,\alpha ,\beta \right),$$

so that

$$\partial_{t}J={\left(\partial_{\alpha }w_{m}\times e_{\beta }+e_{\alpha }\times \partial_{\beta }w_{m}\right)}^{T}{\vec{n}}_{m}.$$

Write $w_{m}=\rho_{m}+\zeta_{m}{\vec{n}}_{m}$ so that

$$\partial_{t}J={\left(\partial_{\alpha }\rho_{m}\times e_{\beta }+e_{\alpha }\times \partial_{\beta }\rho_{m}\right)}^{T}{\vec{n}}_{m}+\zeta_{m}{\left(\partial_{\alpha }{\vec{n}}_{m}\times e_{\beta }+e_{\alpha }\times \partial_{\beta }{\vec{n}}_{m}\right)}^{T}{\vec{n}}_{m}.$$

One has

$${\vec{n}}_{m}^{T}\left(\partial_{\alpha }\rho_{m}\times e_{\beta }+e_{\alpha }\times \partial_{\beta }\rho_{m}\right)=J\left(\text{di}v_{S\left(t\right)}\rho_{t}\right)\left(\psi_{m}\left(t,\cdot \right)\right)$$

and

$${\vec{n}}_{m}^{T}\left(\partial_{\alpha }{\vec{n}}_{m}\times e_{\beta }+e_{\alpha }\times \partial_{\beta }{\vec{n}}_{m}\right)=-2JH_{S\left(t\right)}\left(\psi_{m}\left(t,\cdot \right)\right).$$

These identities can be proved from (4) and (5) by introducing an orthonormal basis $\left(e_{1},e_{2}\right)$ of $T_{\psi_{m}\left(t,x\right)}S\left(t\right)$ and expanding the left-hand sides as functions of the coordinates of $e_{\alpha }$ and $e_{\beta }$ in this basis (cf. [18], lemma 3.19). Using this, we get

$$\partial_{t}J=J\;\left(\text{di}v_{S\left(t\right)}\rho_{t}-2H_{S\left(t\right)}\right)\circ \psi_{m}$$

from which one deduces that

$\partial_{t}\sigma =\sigma \;\left(\text{di}v_{S(t)}\rho_{t}-2H_{S(t)}\right)\circ \psi$ □

Define the “equivolumetric thickness” along the streamline starting at x by

(6) $$\gamma \left(t,x\right)=\int_{0}^{t}\partial_{t}\psi {\left(u,x\right)}^{T}\vec{n}\left(u,x\right)\sigma \left(u,x\right)\;du.$$

A strict interpretation of Bok's hypothesis requires that this expression does not depend on x, i.e., that there exists a function t↦λ(t) such that

(7) $$\partial_{t}\psi {\left(t,x\right)}^{T}\vec{n}\left(t,x\right)\sigma \left(t,x\right)=\lambda \left(t\right)$$

for all $x\in S_{0}$. (In which case $\gamma \left(t,x\right)=\int_{0}^{t}\lambda \left(u\right)du$.) One can, without loss of generality, assume that λ does not depend on t, as this can be achieved by applying a time change to the evolution. More precisely, one can replace ψ by $\tilde{\psi }$ such that $\tilde{\psi }\left(\tau (t),x\right)=\psi \left(t,x\right)$ with

$$\tau \left(t\right)=\frac{\int_{0}^{t}\lambda \left(u\right)du}{\int_{0}^{1}\lambda \left(u\right)du}$$

in which case $\tilde{\psi }$ satisfies (7) with constant right-hand side

$$\tilde{\lambda }=\int_{0}^{1}\lambda \left(u\right)du.$$

Therefore, assuming that λ is constant, we now apply Proposition 1 to obtain a surface propagation equation that is equivalent to (7). We will decompose $w=\partial_{t}\psi \circ \psi^{-1}$ in the form

$$w\left(y\right)=\rho \left(y\right)+\frac{\lambda }{\sigma \left(\psi^{-1}\left(y\right)\right)}N\left(y\right),$$

as required by (7). One then has the following proposition.

Proposition 2 *A laminar coordinate system*ψ*satisfies Bok's hypothesis if there exists a vector field*ρ*tangent to the layers defined by*ψ*and a constant*λ*such that*

(8) $$\left\{ \begin{array}{c} \partial_{t}\psi \left(t,x\right)=\rho \left(\psi \left(t,x\right)\right)+\frac{\lambda }{\sigma \left(t,x\right)}\vec{n}\left(t,x\right)\\ \partial_{t}\sigma \left(t,x\right)=\sigma \left(t,x\right)\text{di}v_{S\left(t\right)}\rho \left(\psi \left(t,x\right)\right)-2\lambda H\left(t,x\right) \end{array}\right.$$

with ψ(0,x)=x and σ(0,x)=1 for all $x\in S_{0}$.

Eq. (8) provides an evolution equation controlled by ρ (such that at all times, ρ(t,·) is a vector field on the evolving surface S(t)) whose solution satisfies the equivolumetric hypothesis. A detailed study of this system of equations (including its well-posedness for a given choice of ρ), and of the optimal control problem consisting in optimizing ρ with the constraint that $S\left(1\right)=S_{1}$ are challenging open problems that will not be addressed in this paper. Even if possible, it would also be counter-intuitive to require a constant equivolumetric thickness, γ(1,x), in (6). So, in the next section, we discuss a solution to a simpler problem that we refer to as a “localized” Bok's hypothesis.

#### Localized Bok's hypothesis

In this section, we replace the strong constraint (7) by a weaker one

(9) $$\partial_{t}\psi {\left(t,x\right)}^{T}{\vec{n}}_{S\left(t\right)}\left(\psi \left(t,x\right)\right)\sigma \left(t,x\right)=\lambda \left(t\right)c_{0}\left(x\right)$$

for a given function $c_{0}$. Here again, there is no loss of generality in assuming that λ is constant, and, including if needed this constant in $c_{0}$, in taking λ=1 (so that $c_{0}$ coincides with the equivolumetric thickness γ(1,x)). Proposition 2 can be directly extended in this setting.

Proposition 3 *A laminar coordinate system*ψ*satisfies the localized form of Bok's hypothesis for a given equivolumetric thickness*$c_{0}$*if there exists a vector field*ρ*tangent to the layers defined by*ψ*such that*

(10) $$\left\{ \begin{array}{c} \partial_{t}\psi \left(t,x\right)=\rho \left(\psi \left(t,x\right)\right)+\frac{c_{0}\left(x\right)}{\sigma \left(t,x\right)}\vec{n}\left(t,x\right)\\ \partial_{t}\sigma \left(t,x\right)=\sigma \left(t,x\right)\text{di}v_{S\left(t\right)}\rho \left(\psi \left(t,x\right)\right)-2c_{0}\left(x\right)H\left(t,x\right) \end{array}\right.$$

with ψ(0,x)=x and σ(0,x)=1 for all $x\in S_{0}$.

Because the function $c_{0}$ can be chosen freely, (9) (or a solution of system (10)) is much easier to obtain while satisfying the condition $\psi \left(1,S_{0}\right)=S_{1}$ and we now show that it can be achieved starting from any laminar coordinate system ψ by applying a space-dependent time change. Indeed, let $\psi_{\tau }\left(\tau (t,x),x\right)=\psi \left(t,x\right)$, where, for each $x\in S_{0}$, t↦τ(t,x) is an increasing differentiable function from [0,1] onto [0,1]. Then, introducing as above local coordinates α and β on $S_{0}$, we have

$$\partial_{\alpha }\psi \left(t,x\right)=\partial_{\alpha }\tau \;\partial_{t}\psi_{\tau }\left(\tau ,x\right)+\partial_{\alpha }\psi_{\tau }\left(\tau ,x\right)$$

$$\partial_{\beta }\psi \left(t,x\right)=\partial_{\beta }\tau \;\partial_{t}\psi_{\tau }\left(\tau ,x\right)+\partial_{\beta }\psi_{\tau }\left(\tau ,x\right)$$

$$\partial_{t}\psi \left(t,x\right)=\partial_{t}\tau \;\partial_{t}\psi_{\tau }\left(\tau ,x\right)$$

As a consequence:

$$\vec{n}{\left(t,x\right)}^{T}\partial_{t}\psi \left(t,x\right)\sigma \left(t,x\right)=\text{det}\left(\partial_{t}\psi ,\partial_{\alpha }\psi ,\partial_{\beta }\psi \right)\left(t,x\right)$$

$$=\partial_{t}\tau \left(t,x\right)\;\det\left(\partial_{t}\psi_{\tau },\partial_{\alpha }\psi_{\tau },\partial_{\beta }\psi_{\tau }\right)\left(\tau ,x\right)$$

$$=\partial_{t}\tau \left(t,x\right)\;{\vec{n}}_{\tau }{\left(\tau ,x\right)}^{T}\partial_{t}\psi_{\tau }\left(\tau ,x\right)\sigma_{\tau }\left(\tau ,x\right).$$

In order that (9) holds for $\psi_{\tau },$ we therefore need to define τ so that (for some function $c_{0}$)

$$c_{0}\left(x\right)\partial_{t}\tau \left(t,x\right)=\vec{n}{\left(t,\psi \left(t,x\right)\right)}^{T}\partial_{t}\psi \left(t,x\right)\sigma \left(t,x\right)$$

In order to have τ(0,m)=0, τ(1,m)=1, we need

$$c_{0}\left(x\right)=\int_{0}^{1}\vec{n}{\left(u,\psi \left(u,x\right)\right)}^{T}\partial_{t}\psi \left(u,x\right)\sigma \left(u,x\right)\;du$$

and then

(11) $$\tau \left(t,x\right)=\frac{1}{c_{0}\left(x\right)}\int_{0}^{t}\vec{n}{\left(u,\psi \left(u,x\right)\right)}^{T}\partial_{t}\psi \left(u,x\right)\sigma \left(u,x\right)\;du.$$

So, the time change is provided by the relative volumetric depth along the streamlines (that are left unchanged in the operation). The equivolumetric layers at level ϵ are provided by points ψ(t,x) along the streamlines satisfying τ(t,x)=ϵ (in the original parametrization).

#### Numerical implementation

Because they rely on level sets, the two recent approaches [3,4] (described in supplementary material) are Eulerian, i.e., they work in the 3D volume Ω and layers are isosurfaces associated with scalar functions defined on Ω while streamlines are integrated using the gradient of these functions.

Our approach is different in that it directly models layers as parametrized surfaces $S\left(t\right)=\psi \left(t,S_{0}\right)$, so that our implementation is based on a triangulation of $S_{0}$ and a discretization of the time interval. The streamlines are obtained as solutions of the ordinary differential equation (ODE) $\partial_{t}y=v\left(t,y\right)$, where v is obtained from the LDDMM algorithm and provided by (2). Importantly, this time-dependent vector field is discretized in time only, and known analytically as a function of y. In particular, its space derivatives can be evaluated without approximation. As above, it specifies a flow of diffeomorphisms of $R^{3}$ through the equation

$$\partial_{t}\varphi \left(t,x\right)=v\left(t,\varphi \left(t,x\right)\right)$$

with φ(0,x)=x for all $x\in R^{3}$.

The surface Jacobian σ can be evaluated using the evolving triangulated surfaces: if x is a vertex on $S_{0}$, we let a(0,x) denote the area of the one-ring centered at x (the union of all triangles that contain x). Similarly, we let a(t,x)denote the area of the one-ring around φ(t,x) in the triangulated surface $S\left(t\right)=\varphi \left(T,S_{0}\right)$ (which has the same triangle structure as $S_{0}$). On can then define

$$\sigma \left(t,x\right)=\frac{a\left(t,x\right)}{a\left(0,x\right)}.$$

This is the approximation that is used in our simulations, and it is accurate provided that the triangulation of $S_{0}$ is fine enough without flat triangles. An alternative procedure is also possible, since one has, in this context

(12) $$\sigma \left(t,x\right)=\text{det}\left(\partial_{x}\varphi \left(t,x\right)\right)\left|\partial_{x}\varphi {\left(t,x\right)}^{-T}\nu \left(0,x\right)\right|$$

for $x\in S_{0}$. (The “−T” exponent refers to the inverse of the transpose matrix.) Recall that φ(t,·) is (for fixed time t) a diffeomorphism of $R^{3}$, hence defined on the whole space (unlike ψ, which, for laminar coordinates, is only defined on $S_{0}$, and in this special case, is defined as the restriction of φ to this surface). This implies that $\partial_{x}\varphi \left(t,x\right)$ is a 3×3 matrix. To prove (12) one can just notice that, in a local chart m

$$\sigma \left(t,x\right)=\frac{\left|\left(\partial_{x}\varphi \left(t,x\right)\partial_{\alpha }m\right)\;\times \;\left(\partial_{x}\varphi \left(t,x\right)\partial_{\beta }m\right)\right|}{\left|\partial_{\alpha }m\times \partial_{\beta }m\right|}$$

and use the fact that for any matrix A and vector u and v, one has $Au\times Av=\text{det}\left(A\right)A^{-T}\left(u\times v\right)$.

The time evolution of the vector $\zeta \left(t,x\right)=\text{det}\left(\partial_{x}\varphi \left(t,x\right)\right)\partial_{x}\varphi {\left(t,x\right)}^{-T}\nu \left(0,x\right)$ is provided by

$$\partial_{t}\zeta =\text{div}\left(v\right)\left(\varphi \left(t,x\right)\right)-\partial_{x}v\left(\varphi \left(t,x\right)\right)\zeta \left(t,x\right)\;.$$

This can be integrated along streamlines using the expression of v in (2), from which, as mentioned, space derivatives can be evaluated exactly.

## Method validation

**Fig. 5:**
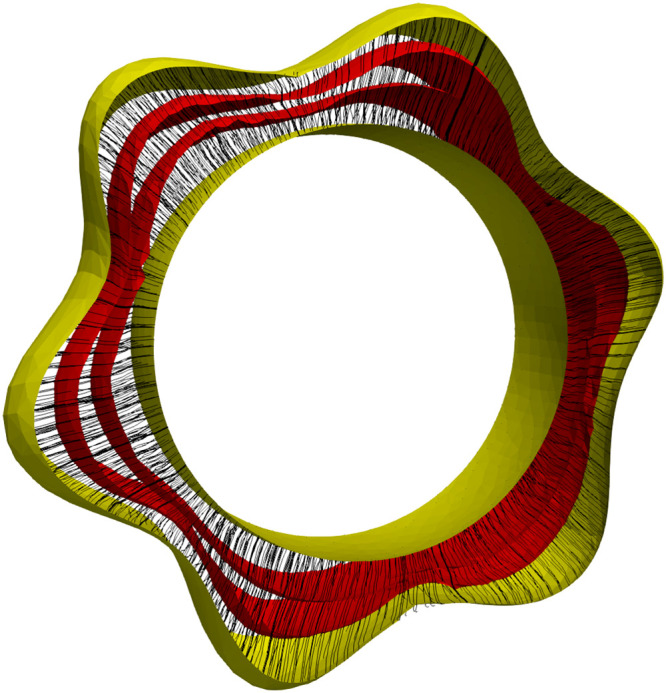
Estimated equivolumetric layers (in red) at times t=0.3 and 0.6 for the synthetic example of Fig. 3.

**Fig. 6:**
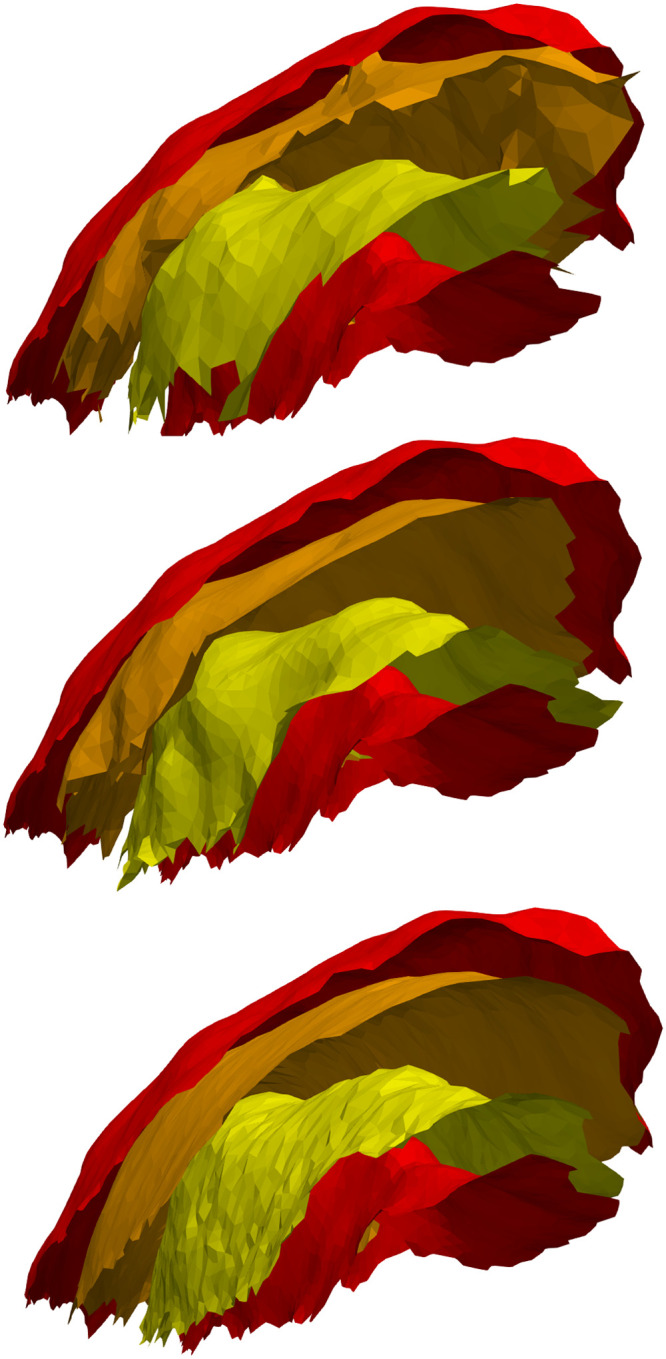
Equivolumetric layers for a marmoset auditory cortex at t=0.25 (orange) and t=0.75 (yellow) using Laplacian [[3], [22]] (top), level set [[4], [21]] (middle) and the proposed method (bottom).

**Fig. 7:**
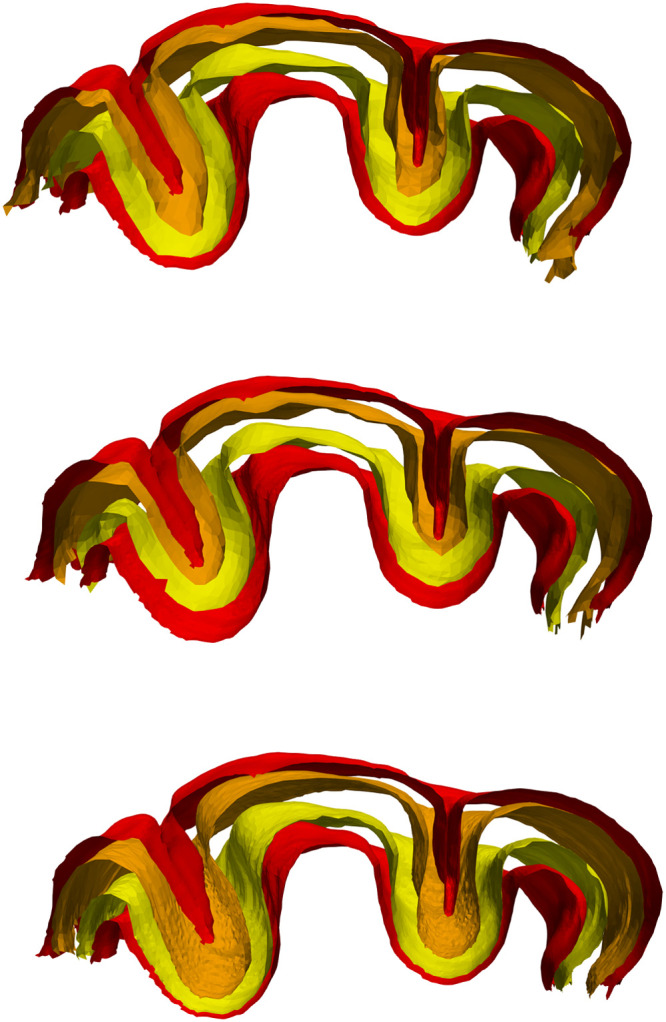
Equivolumetric layers for a feline auditory cortex at t=0.25 (orange) and t=0.75 (yellow) using Laplacian [[3], [22]] (top), level set [[4], [21]] (middle) and the proposed method (bottom).

**Fig. 8:**
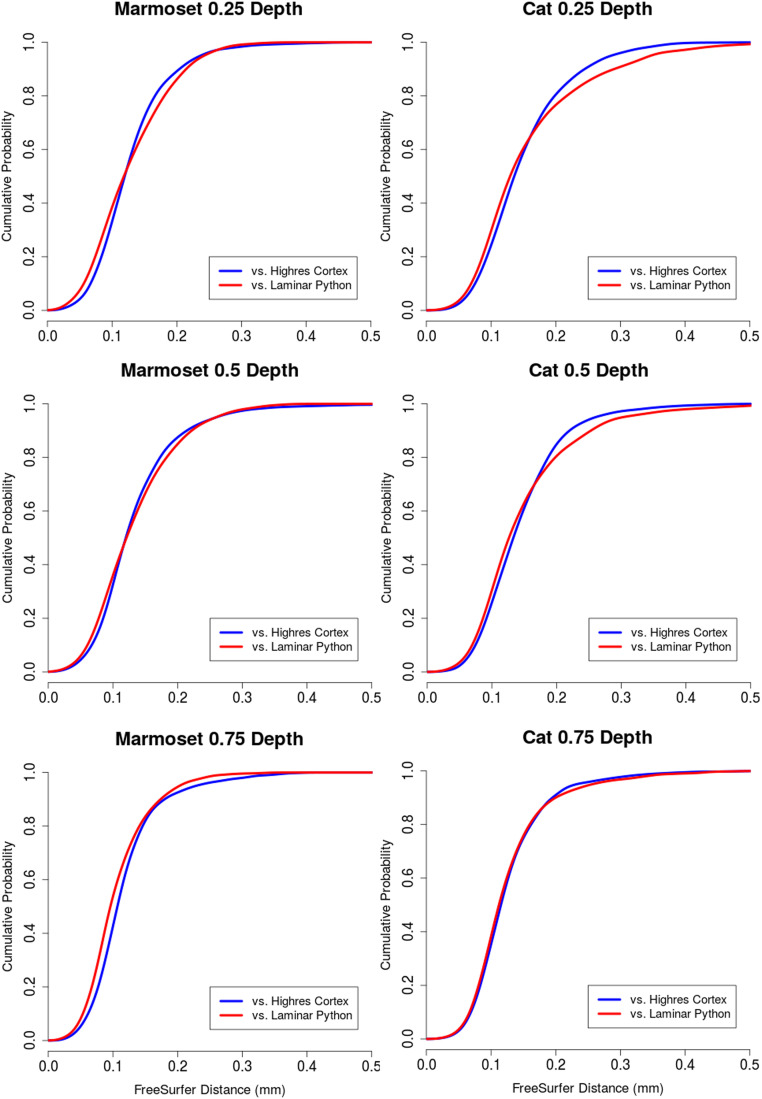
CDFs of FreeSurfer distances of equivolumetric surfaces at t=0.25,0.5,1.0 from Fig. 6, Fig. 7 computed via Github packages - Laplacian (Highres Cortex, [22]) and Level Set(Laminar Python, [21]) - relative to surfaces computed via the proposed method.

Results of the numerical implementation are presented for three cases. Fig. 5 shows the result for the synthetic data of Fig. 3. Fig. 6, Fig. 7 respectively show the results for the marmoset auditory cortex (obtained from [19]) and feline auditory cortical regions (obtained from [20]). Here, the proposed method is compared with those of Waehnert et al. [4] and Leprince et al. [3] computed via Github packages [21,22]. Fig. 8 shows the corresponding cumulative distribution of distances of equivolumetric surfaces at t=0.25,0.5,1.0 relative to those via the proposed method. Here, for surfaces $S_{1}$ and $S_{0}$ with vertices $x_{i}\in S_{1}$ and $y_{j}\in S_{0}$, the distance at the i^th^ vertex of $S_{1}$ is $d_{i}=\frac{1}{2}\left(d\left(x_{i},y_{n}\right)+d\left(x_{m},y_{n}\right)\right)$ where $n={\arg\min d(x_{i},x_{j})\;}_{j}$and $m={\arg\min d(x_{k},x_{n})\;}_{k}$. This FreeSurfer distance [23] returns a value for every vertex and making it more robust against outliers that arise with the Hausdorff distance.

Granted that in the marmoset the auditory cortex is the only area that is folded with a gyral crown, the protrusions of the upper layers seen with the method of Leprince et al. [3] may be attributed to the diverging normal vector fields as one approaches the outer surface. This divergence warrants finer discretization which may be computationally expensive. The primary and higher-order auditory cortical regions in the cat reveals greater differences between the three methods. The t=0.25 layer appears to be closer to the sulcal fundi for Leprince et al. and our methods; in contrast the t=0.75 layer appears to be closer to the gyral crowns for Waehnert et al. and our methods. These differences can be quantified via a distance metric (Fig. 8) and may be attributed to the representation of curvature–direct or indirect–in the computations. Future work will examine how disease and disorder affect the equivolumetric depths of layers, pyramidal cells and other cortical elements in 3D. This will build upon previous work in 2D [20]. Our laminar coordinate system also has a straightforward application to cortical layer segmentation. As a prior, it could help distinguish between layers of similar microanatomy (e.g., cortical layers III and V) that cannot be separated using staining intensity alone.

## Limitations

The time change introduced to reparametrize the coordinates in order to make them compliant with Bok's hypothesis leaves the streamlines invariant while changing the layers. As a consequence, if streamlines were perpendicular to the layers to start with, this property is generally lost after reparametrization. Finding an equivolumetric coordinate system with perpendicular streamlines is a significantly more arduous problem. Using Proposition 3, in which one must set ρ=0, one sees that this problem requires to estimate a scalar field $c_{0}$ on $S_{0}$ such that the solution of (10) satisfies $\psi \left(1,S_{0}\right)=S_{1}$. Whether this inverse problem is both well posed and biologically valid, and whether stable numerical algorithms can be designed to solve it, are open questions that we plan to address in future work.

## Ethics statements

Feline experiments were approved by the local state authorities and were performed in compliance with the guidelines of the European Community European Union legislation for the care and use of laboratory animals (EU Directive 2010/63/EU), ARRIVE guidelines and the German Animal Welfare Act (TierSchG).

## CRediT authorship contribution statement

**Laurent Younes:** Conceptualization, Methodology, Software, Visualization, Validation, Writing – original draft. **Kwame S. Kutten:** Visualization, Data curation, Validation, Writing – review & editing. **J. Tilak Ratnanather:** Conceptualization, Methodology, Funding acquisition, Writing – original draft.

## Declaration of competing interest

The authors declare that they have no known competing financial interests or personal relationships that could have appeared to influence the work reported in this paper.

## Data Availability

Data will be made available on request.

## Appendix Supplementary materials

Image, application 1
