# Supplementary material for: Normal and equivolumetric coordinate systems for cortical areas
Source: MethodsX. 2024 Apr 1;12:102689. doi: 10.1016/j.mex.2024.102689 (PMC11022084; doi:10.1016/j.mex.2024.102689)
Supplement: Supplementary file 1 [file mmc1.docx]

**Supplementary material *and/or* additional information**

*Level Set Methods*

Assume that $\Omega\subset\mathbb{R}^{3}$ and that its boundary has two connected components, providing $S_{0}$ and $S_{1}$, which must therefore be closed surfaces. The typical application is when $\Omega$ is the region between two non-intersecting nested sphere-like surfaces.

Assume that one is given a $C^{2}$ submersion $F:\bar{\Omega}\to[0,1]$ such that $S_{0}=F^{-1}(0)$ and $S_{1}=F^{-1}(1)$. (This assumes that $F$ is onto and that $\nabla F(x)\neq0$ for all $x\in\bar{\Omega}$.) Define the vector field

$$v(x)=\nabla F/|\nabla F|^{2}, x\in\Omega$$

and let $\psi(t,x)$, $t\in[0,1]$, $x\in S_{0}$ satisfy

$$\partial_{t}\psi(t,x)=v(\psi(t,x))$$

with $\psi(0,x)=x$, so that $\psi$ is the restriction to $S_{0}$ of the flow associated with the vector field $v$. Then, one has

$$\partial_{t}F(\psi(t,x))=\nabla F(\psi(t,x))^{T}v(\psi(t,x))=1$$

which implies that $F(\psi(t,x))=t$ for all $x\in S_{0}$ and $t\in[0,1]$. The function $\psi:[0,1]\times S_{0}\to\bar{\Omega}$ provides a laminar coordinate system of $\bar{\Omega}$ as defined above. The thickness along streamlines is in particular defined by

$$\theta(x)=\int_{0}^{1} |\nabla F(\psi(t,x))|^{-1} dx.$$

Notice that, since the layers $S_{t}$ coincide with the level sets of $F$, the vector field $v$ (and therefore the streamlines) are necessarily perpendicular to them.

Given $S_{0}$ and $S_{1}$, one therefore needs to define a suitable function $F$. Jones et al. [5] defined $F$ as the solution of the Laplace equation $\Delta F=0$ with boundary conditions $F=0$ on $S_{0}$ and $F=1$ on $S_{1}$. Similarly, Waehnert et al. [8] proposed a construction starting with level-set representations of $S_{0}$ and $S_{1}$, represented by two functions $U_{0}$ and $U_{1}$ such that $S_{i}=U_{i}^{-1}(0)$, $i=0,1$. This leads to the definition

|  | $F(t,\cdot)=t+\mathcal{S}((1-\rho)U_{0}+\rho U_{1})$ | (13) |
| --- | --- | --- |

where $\mathcal{S}$ is a smoothing operator based on a topology-preserving mean-curvature motion equation. More precisely, given a function $\bar{U}$, the smoothed function, $\mathcal{S}\bar{U},$ is obtained as the limit when time $s\to\infty$ of the solution $U(s,\cdot)$ of

$$\partial_{s}U+(U-\bar{U})|\nabla U|=\epsilon|\nabla U|\mathrm{div}\left( \frac{\nabla U}{|\nabla U|} \right) .$$

*Diffeomorphic Volume Mapping*

Das et al. [3] assumed that the surfaces $S_{0}$ and $S_{1}$ are represented as boundaries of two open set $\Omega_{0}$ and $\Omega_{1}$ (so that $S_{0}=\partial\Omega_{0}$ and $S_{1}=\partial\Omega_{1}$) with $\bar{\Omega}_{0}\subset\Omega_{1}$. Then a variant [1] of the large deformation diffeomorphic metric mapping (LDDMM) algorithm [2] was used to estimate a flow of diffeomorphisms $\varphi(t,\cdot):\mathbb{R}^{3}\to\mathbb{R}^{3}$, $t\in[0,1]$ such that $\varphi(1,\Omega_{0})\simeq\Omega_{1}$. The function $\psi$ can then be defined as the restriction of $\varphi$ to $[0,1]\times S_{0}$.

In a similar spirit, Fischl and Sereno [4] build a laminar coordinate system by estimating a vector field $v$ between $S_{0}$ and $S_{1}$. This vector field is optimized as a minimizer of two energy terms, a first one ensuring that $v$ is almost normal to the two surfaces, and a second one acting like a regularizer, roughly equivalent to a second-order Sobolev norm applied to $v$ along the layers (this equivalence requires some interpolation, as this approach only provides a discrete, discretization-dependent, objective function).

## Interpretation of recent models

We now interpret two recent attempts to model Bok’s hypothesis [7, 8] in our framework.

Waehnert et al. [8] start with streamlines estimated using (13). Equivolumetric layers are then estimated using the same equation, replacing the constant value $\rho$ by a function $\rho(x)$, that is determined as follows. Using our notation, one first assumes that

$$\sigma\left( t,x \right)=\left( 1-t \right)+t\sigma\left( 1,x \right),$$

therefore making a linear approximation of the surface change. Here, one takes $t=s/\theta$, where $s$ is the arc length along the streamline and $\theta$ is the thickness (the length of the streamline). For $x\in S_{0}$, the value of $\sigma(1,x)$ is estimated as a function of the curvatures at both ends of the streamline starting at $x$ (refer to [8, 6] for more details and justification). Integrating along streamlines, which are perpendicular to the layers because of the level set formulation, one obtains an expression of the equivolumetric depth given by

$$\mathcal{V}_{x}(\rho)=\theta\int_{0}^{\rho} \sigma(t,x)dt=\theta\rho\left( 1+\frac{\rho}{2}(\sigma(1,x)-1) \right).$$

Given $\epsilon\in[0,1]$, one defines a target level $\rho_{\epsilon}(x)$ corresponding to the layer at equivolume $\epsilon$ by solving $\mathcal{V}_{x}(\rho)=\alpha\mathcal{V}_{x}(1)$, which is a quadratic equation in $\rho$.

Leprince et al. [7] start with a Laplacian-based level-set definition of streamlines [5]. They estimate $\sigma(t,\cdot)$ along the streamlines by solving

$$\partial_{t}\sigma(t,x)=-2\theta(x)\sigma(t,x)H(t,x)$$

which corresponds to (3) for a constant-speed normal evolution $\partial_{t}\psi(t,x)=\theta\left( x \right)\vec{n}(t,x)$. Note that, in the level set approach, one has $\vec{n}(t,x)=(\nabla F/|\nabla F|)(\psi(t,x))$ and $2H(t,x)=-\mathrm{div}(\nabla F/|\nabla F|)(\psi(t,x))$. Equivolumetric layers are then deduced from this computation.

[1] B. AVANTS, C. L. EPSTEIN and J. C. GEE, *Symmetric shape averaging in the diffeomorphic space*, *2007 4th IEEE International Symposium on Biomedical Imaging: From Nano to Macro*, IEEE, 2007, pp. 636-639.

[2] M. F. BEG, M. I. MILLER, A. TROUVÉ and L. YOUNES, *Computing large deformation metric mappings via geodesic flows of diffeomorphisms*, International Journal of Computer Vision, 61 (2005), pp. 139--157.

[3] S. R. DAS, B. B. AVANTS, M. GROSSMAN and J. C. GEE, *Registration based cortical thickness measurement*, NeuroImage, 45 (2009), pp. 867--879.

[4] B. FISCHL and M. I. SERENO, *Microstructural parcellation of the human brain*, NeuroImage, 182 (2018), pp. 219-231.

[5] S. E. JONES, B. R. BUCHBINDER and I. AHARON, *Three-dimensional mapping of cortical thickness using Laplace's Equation*, Human Brain Mapping, 11 (2000), pp. 12--32.

[6] V. G. KEMPER, F. DE MARTINO, T. C. EMMERLING, E. YACOUB and R. GOEBEL, *High resolution data analysis strategies for mesoscale human functional MRI at 7 and 9.4T*, NeuroImage, 164 (2018), pp. 48-58.

[7] Y. LEPRINCE, F. POUPON, T. DELZESCAUX, D. HASBOUN, C. POUPON and D. RIVIÈRE, *Combined Laplacian-equivolumic model for studying cortical lamination with ultra high field MRI (7T)*, *2015 IEEE 12th International Symposium on Biomedical Imaging (ISBI)*, 2015, pp. 580--583.

[8] M. D. WAEHNERT, J. DINSE, M. WEISS, M. N. STREICHER, P. WAEHNERT, S. GEYER, R. TURNER and P. L. BAZIN, *Anatomically motivated modeling of cortical laminae*, NeuroImage, 93 (2014), pp. 210--220.
